# Cut-off values for the applied version of the Beck Depression Inventory in a general working population

**DOI:** 10.1186/s12995-015-0067-4

**Published:** 2015-07-19

**Authors:** Uwe Rose, Stefanie March, Melanie Ebener, Jean-Baptist du Prel

**Affiliations:** Federal Institute of Occupational Safety and Health (FIOSH), Nöldnerstrasse 40-42, 10317 Berlin, Germany; Institute of Social Medicine and Health Economics, Otto-von-Guericke-University of Magdeburg, Leipziger Str. 44, 39120 Magdeburg, Germany; Institute for Safety Engineering, University of Wuppertal, Gaußstr. 20, 42119 Wuppertal, Germany

**Keywords:** Depressive symptoms, Beck Depression Inventory, BDI, Functioning, Employees, Cut-off values, Receiver operating characteristics, Work, Work ability

## Abstract

**Background:**

The Beck Depression Inventory (BDI) for the assessment of depressive symptoms is well established in clinical settings. An applied version (BDI-V) was previously developed in German for use within epidemiologic studies. The current study analyses the association between this applied version of the BDI and different measures of functioning. The aim is to determine BDI-V cut-off values when used in a population of employees.

**Methods:**

The study included 6339 employees of the first wave of a German cohort study on work, age, health and work participation. Depressive symptoms were assessed by an applied version of the BDI-V. Data on functioning were obtained from personal interviews. The determination of cut-off values is achieved with the min-max principle for classification applied to receiver operating characteristic (ROC) curves.

**Results:**

The min-max principle points to a BDI-V cut-off between 20 and 24 for male and between 23 and 28 for female respondents. The corresponding sensitivities range between 0.64 and 0.75 for males and between 0.59 and 0.74 for females. Specificities range between 0.64 and 0.75 for males and between 0.60 and 0.74 for females. Female respondents have higher BDI-V cut-offs for all criteria.

**Conclusions:**

The range of values is lower than a recommendation in a former study. In addition to this, the values differ for gender. The current analyses focus on an easier-to-use version of the BDI formerly applied for epidemiologic studies. The determination of cut-off values is based on criteria which are indicators for impairment in (work) functioning in a population of employees. Therefore, grouping of individuals according to the reported cut-off values is guided by the relevance of these scores for occupational functioning.

## Background

Depressive symptoms are highly relevant outcomes in population based research fields such as occupational epidemiology. They are associated with a high loss of productivity and working time due to sickness absence and early retirement [1, 2]. Therefore instruments which measure depressive symptoms and have been validated in the setting of a general working population are of high value for research, but unfortunately seldom available.

When applying such instruments to epidemiological studies, there are typically constraints on the length of the interview. Therefore, a questionnaire for depressive symptoms is required to be fairly short. With these aspects in mind we investigated the validity of the applied version of the Beck Depression Inventory (BDI-V) which we used in the context of the lidA (leben in der Arbeit; English: living at work)-study: a German cohort study on work, age, health and work participation [3].

The assessment of depressive symptoms by means of a questionnaire is different than the assessment of clinical depression based on a standardised interview. According to the standards of classification systems such as the fourth and fifth editions of the Diagnostic and Statistical Manual of Mental Disorders (DSM) of the American Psychiatric Association [4, 5] a major depressive episode is diagnosed by a standardised interview. The diagnosis is based on a set of diagnostic criteria, and the application of a rule for classification. Starting with depressed mood and loss of interest or pleasure, the patient is asked about a pattern of symptoms (i.e. changes in appetite, sleep disturbances etc.). A further criterion for an episode is met when the symptoms cause clinically significant distress or impairment in important areas of functioning.

In contrast to the assessment by a clinician using specific criteria there is a long-standing tradition of self-reported measures of depressive symptoms. One example is the BDI. Respondents have to choose one statement from a list of four which best describes how they have been feeling during the past week up to and including the date of assessment. The aggregation of item responses to a score allows for an assessment of depression symptoms severity. A higher score might be an indication for a greater impairment in different areas of life. But this association is only an assumption and is not based on the direct assessment of “an impairment in social, occupational, or other important areas of functioning” in the DSM-V (p.161, [5]).

This DSM criterion is very similar to “activity limitations” as described in the International Classification of Functioning, Disability, and Health (ICF) coordinated by the WHO [6]. The classification system of the ICF goes beyond the diagnosis of particular patterns of symptoms and signs: It focuses on the effects of a health condition on the person and her functioning [7–9]. These effects are classified by performance in daily activities and by participation in life situations, while considering the environmental and personal factors that affect psychological health [7–9]. In this sense, self-report measures are narrowed in their assessment to symptomatology and do not provide a direct link to consequences for a person’s daily functioning. Such reduction to symptomatology as found in typical self-reporting instruments is not a hidden fault or shortcoming. It is rather a prerequisite for establishing the validity of scores in depression inventories by appraising the evidence with regards to their relation to other relevant criteria. This procedure for a validation is in line with “The Standards for Educational and Psychological Testing” [10], which describe several sources of evidence to illuminate different aspects of validity even in the absence of a gold standard for testing.

The starting point for the current study is an applied version of the original BDI [11, 12]. This instrument is well established in clinical settings. It has been modified for applications in survey research. Criterion-referenced validity of this applied version of the BDI (German BDI-V) was investigated [13] using correlations between the BDI-V and similar or convergent self-rating scales of depression. Moreover, Schmitt et al. [13] compared the scores between different clinical and nonclinical samples. This kind of evidence supports the assumption that all these instruments are similar representations of the same concept (depression). But the relationship between BDI-V and other external criteria like general functioning in relevant areas of life remains unclear, whereas both aspects are part of standardised clinical interviews. Therefore, the current analysis will examine these aspects of general functioning and work ability, including their association with the BDI-V scores, in a sample of middle-aged employees. The main aim is the determination of BDI-V cut-off values with reference to functioning.

## Methods

### Design

lidA is a German prospective cohort study in which individuals will be followed up in three year intervals. The study investigates the associations of work, health and employment in an ageing work force. The current validation study is based on respondents from the first wave. The focus was on the association between depressive symptoms and functioning. The responses regarding functioning were obtained by a computer assisted personal interview (CAPI). Depressive symptoms were assessed by the BDI-V (see below). The BDI-V used was a paper and pencil version returned to the interviewer in a closed envelope.

### Sample

Employees were recruited in the frame of the first wave of the German lidA-cohort study, which targets work, age, health and work participation in employees born in 1959 and 1965. Forming part of the German baby-boom generation, these employees are highly relevant for lidA’s primary research goal [3]. The response rate was 27.3 percent. The sample was selected in a two stage random process: First 222 sample points from all over Germany were randomly chosen. Second, 6585 study participants were randomly selected from the database of the Institute for Employment Research (IAB) of the Federal Employment Agency on the reference date 31th December 2009. This data, also referred to as Integrated Employment Biographies (IEB), includes all German employees subject to social insurance contributions. Persons who are unemployed, self-employed, freelancers as well as civil servants are excluded by definition [3].

Following the International Labour Organization (ILO), an employee is defined as a person who works at least one hour a week [14]. Thus 6339 employees were available for this analysis.

In Germany, there are extensive legal requirements for data protection. Therefore, an application process in accordance with German social legislation was required to carry out the sampling procedure [15]. The approval was issued by the ethics commitee of the University of Wuppertal.

### Measurements

#### Depressive symptoms

In the original version of the BDI depressive symptoms are assessed by 21 typical symptoms, each with four statements ranked according their severity level and resulting in 84 statements. The wording of items and comprehensiveness of the questionnaire’s structure might be well suited for a clinical setting, but the applicability of the BDI in epidemiological studies is limited by the length of the instrument. This was part of the motivation behind constructing an applied version of the BDI in German by Schmitt et al. [13]. For this reason, only 20 items from the original BDI were selected. One item about weight loss was dropped. Instead of the original BDI consisting of four statements for each symptom, only one statement is used in the simplified version. This sole statement serves as a reference point when participants are asked to rate their frequency of experience by using a simple six-level scale. The range of the sum scores goes from 0 to 100. Schmitt et al. [13] compared the reliability of the original BDI and their simplified version: Cronbach’s alpha of the original version was lower (α = 0.84) than that of the simplified version (α = 0.94). The simplified BDI and the original version correlated with r = 0.83. This high degree of convergence supported by the results of a confirmatory factor analysis showed only a slight deviation from perfect measurement equivalence, in spite of the higher efficiency of the applied BDI-V. Moreover, norm values are provided by Schmitt et al. [16] based on a sample from the general population in Germany. Criterion-referenced validity was investigated [13] by correlations between BDI-V and similar or convergent self-rating scales of depression and by comparing the score of BDI-V between different clinical and nonclinical samples. This kind of evidence supports the assumption that all these instruments are similar representations of the same concept (depression).

#### Functioning

Two items of the Work Ability Index [17] were selected in order to define dichotomous criteria for functioning at work:

- The respondents are asked “How do you rate your current work ability with respect to the physical demands of your work?” (Work ability - physical), and
- “How do you rate your current work ability with respect to the mental demands of your work?” (Work ability - mental)

The response categories for both items are “very good”, “rather good”, “moderate”, “rather poor”, “very poor”. The cut-off is made between “moderate” and “rather poor”.

Three items of the CAPI were extracted from the modified German version of the SF-12 [18]. They are related to mental health, emotional problems and social limitations: “During the last four weeks, how often did you feel that due to …”

- “mental health or emotional problems you achieved less than you wanted to at work or in everyday activities?” (SF-12 role emotional 1),
- “mental health or emotional problems you carried out your work or everyday tasks less thoroughly than usual?” (SF-12 role emotional 2),
- “physical or mental health problems you were limited socially, that is, in contact with friends, acquaintances, or relatives?” (SF-12 social functioning).

The response categories for these three items are “always”, “often”, “sometimes”, “almost never”, and “never”. The cut-off was made between “sometimes” and “almost never”.

### Statistical analysis

**Fig. 1:**
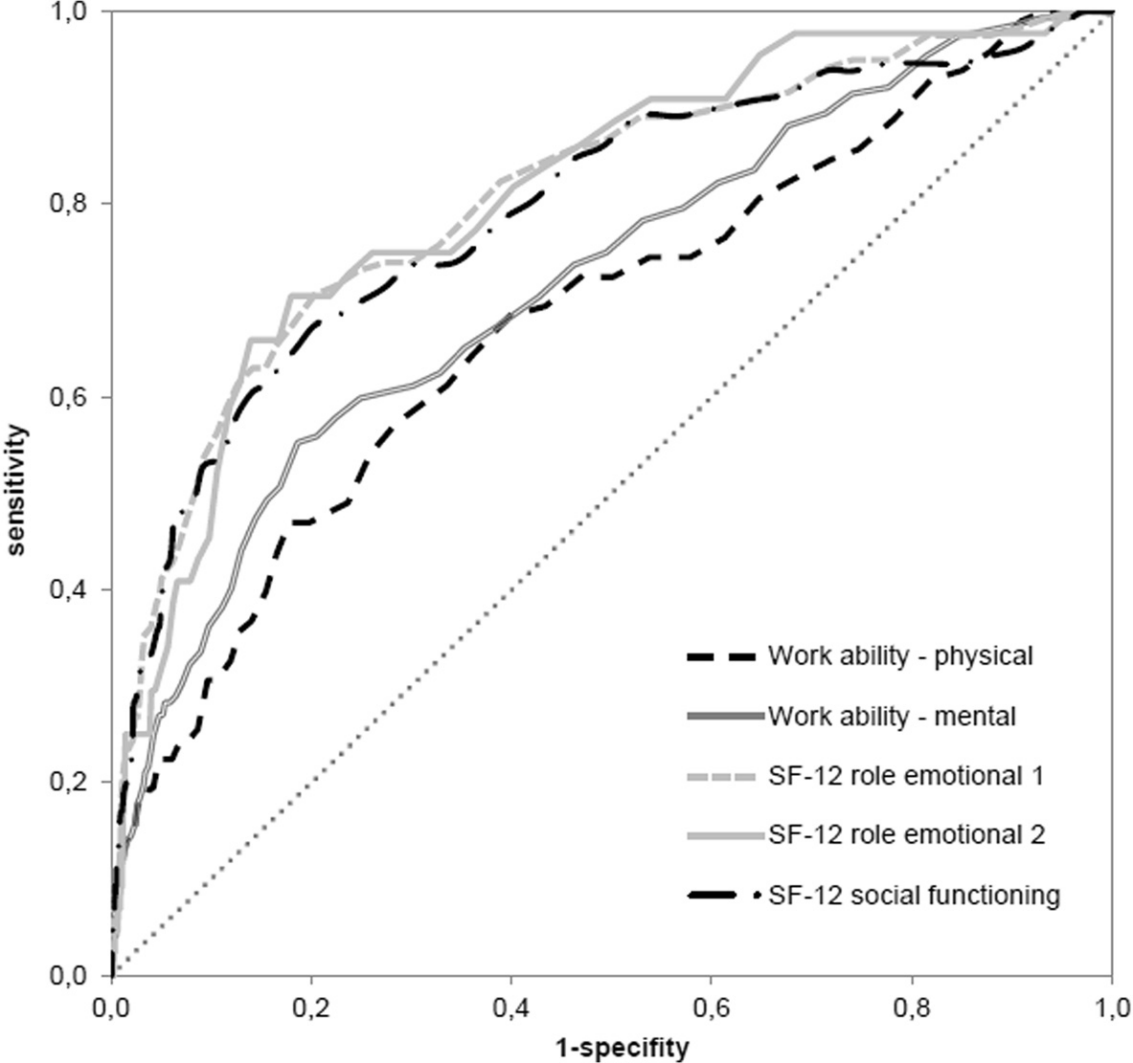
Area Under the Curve for male; sensitivity and 1-Specificity for all BDI-V scores

**Fig. 2:**
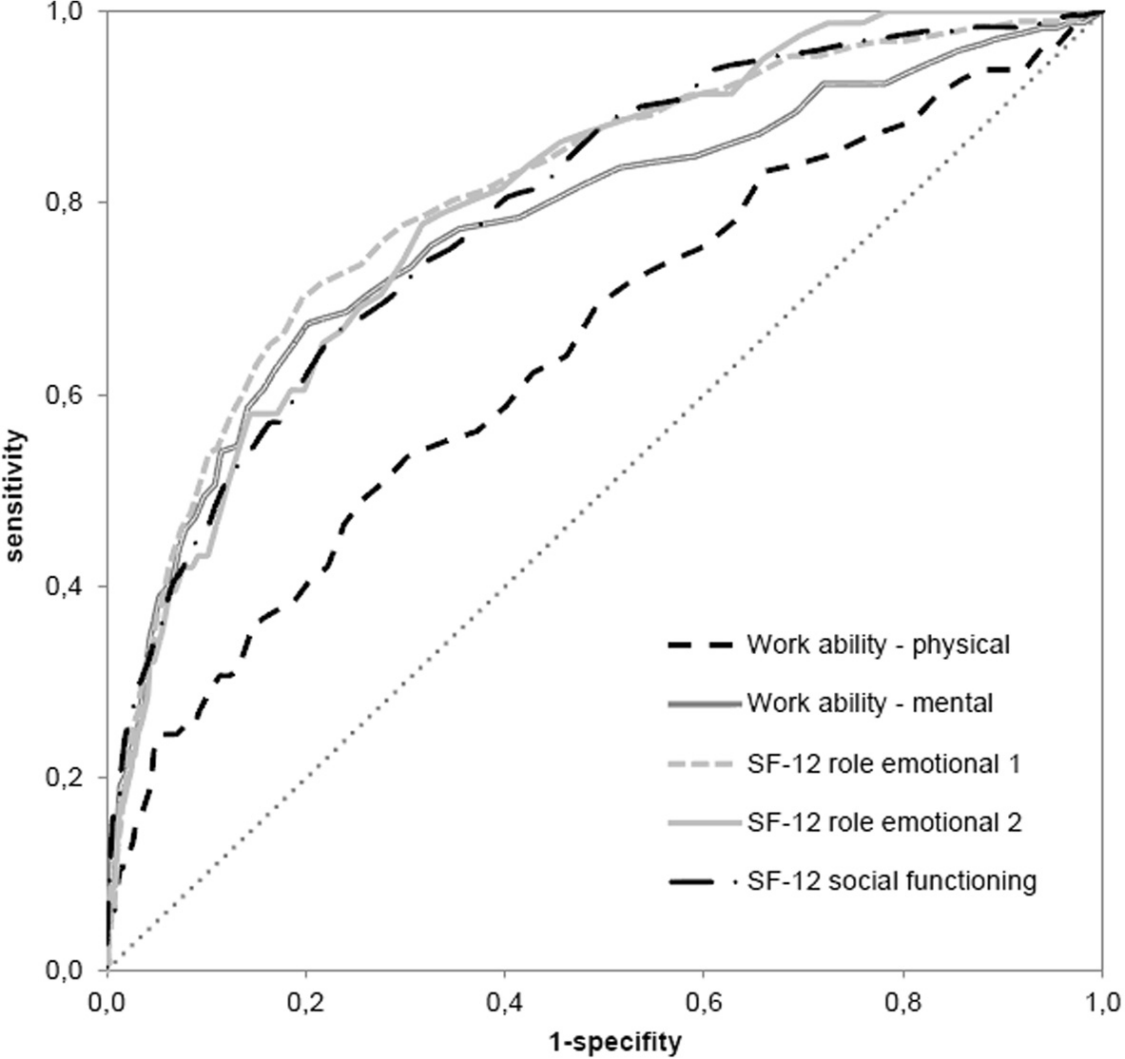
Area Under the Curve for female; sensitivity and 1-Specificity for all BDI-V scores

Criterion referenced validity of the BDI-V was assessed by the rank correlation between BDI-V score and the values of five indicators of functioning. Receiver operating characteristic (ROC) analysis was used to define cut-off values for the BDI-V (Figs. 1 and 2). ROC curves originate from signal detection theory and were later introduced in medicine to validate and improve diagnostic measures [19]. In the classical paradigm for evaluating test performance, the result of an index test is compared with a reference test, typically an instrument widely accepted as a gold standard. An example is liver biopsy as the standard for evaluating liver fibrosis. Such a gold standard as this is to date not available for depressive symptoms. There are however in the field of psychometrics other strategies to evaluate the validity of an index test [10]. An important source for interpretations and inferences of validity is evidence based on relations to other variables. The current analysis focusses on the relationship of the BDI-V to other variables relevant to the participants’ daily functioning and ability to work. ROC curve analysis is then carried out on the basis of these other variables serving as external criteria. BDI-V values were chosen for optimally distinguishing cases with impaired functioning in different areas of life. Participants who have a sum-score above a given cut-off value are cases with impairment. Those below are non-cases. Within a range from 0 to 100 there are 100 possible cut-off values each corresponding to a cumulative percentage. This cumulative distribution is used for the computation of sensitivity (SENS) and specificity (SPEC). The misclassification of cases and non-cases is given by 1 – sensitivity (1 – SENS) and 1 – specificity (1 – SPEC). The area under the curve (AUC) indicates the accuracy of the instrument to detect impairment. It is generated by plotting 1 – SPEC against SENS (see e.g. [19]). There are different strategies and rules to determine the optimal cut-off and to reduce the risk for misclassification (1 – SENS, 1 – SPEC). One common approach is the computation of the Youden-Index (Y): Y = SENS + SPEC – 1 for each possible cut-off. The optimal cut-off is then indicated by the highest Y-value. This rule however allows low values of SENS to be compensated by high values of SPEC and vice versa. Such a compensation is avoided by computing the maximum of both errors in classification. The subsequent selection of a cut-off is based on a minimization of these maximum errors (min-max principle) [20].

## Results

### Sample characteristics

All participants were born in either 1959 or 1965. The predominant employment status among men in these birth cohorts is full time employment (94.7 %), only 3.3 % of the male employees worked part-time (Table 1). The latter is more frequent among women (45.8 %) while 43.4 % were in full-time work. Changes in the employment status (unemployment etc.) are possible due to the time lag between the sampling of addresses and the date of interview in the field. On the other hand, the percentages for the remaining categories (including less than part-time work) are 2.0 % for male and 10.8 % for female respondents. The most prevalent category for the highest school leaving qualification is intermediate qualification with a higher percentage among female (48.0 %) than male respondents (34.7 %). Civil servants, self-employed, and freelancers were excluded from the sampling process. The distribution for net income on this background is characterized by low (male 4.3 %, female 38.1 %) and middle income (male 37.5 %, female 43.8 %).

**Table 1:** Sample characteristics

Variable		Males		Females	
	n	2,552	47.2 %	2,860	52.8 %
Highest school leaving graduation	Low graduation	781	30.6 %	544	19.0 %
	Middle graduation	886	34.7 %	1,374	48.0 %
	High graduation	837	32.8 %	899	31.4 %
	Other/w.o. graduation	51	2.0 %	309	10.8 %
Employment status	Full time	2,417	94.7 %	1,242	43.4 %
	Part time	84	3.3 %	1,309	45.8 %
	Other	51	2.0 %	309	10.8 %
Personal net income	<1000 Euro	109	4.3 %	1,091	38.1 %
	1000 - <2000 Euro	958	37.5 %	1,254	43.8 %
	2000 - <3000 Euro	838	32.8 %	312	10.9 %
	3000 - <4000 Euro	287	11.2 %	49	1.7 %
	≥4000 Euro	242	9.5 %	25	0.9 %
	Missing data	118	4.6 %	129	4.5 %

The median value for BDI-V among males is 16 (central 95 %: 0–50) and among females 20 (central 95 %: 1–56). Both age cohorts are represented by the same median value (18). The lowest value within the central 95 % of the BDI-V distribution for both age cohorts is 1, whereas the highest values are 52 and 55 for 1959 and 1965 cohorts, respectively.

### Analysis of the BDI-V scores

Among male participants the correlation of the BDI-V scores with five criteria was computed using Kendall Tau_b_. Higher values for this rank correlation were found for three criteria: -0.37 for impairment of everyday activities (SF-12 role emotional 1), -0.35 for impairment of everyday tasks (SF-12 role emotional 2), -0.35 for impairment of social functioning (SF-12 social functioning). Smaller (positive) correlations were found for the physical dimension of work ability (Work ability - physical) (0.21) and the mental dimension (Work ability - mental) (0.30).

The correlations among female participants were -0.39 (SF-12 role emotional 1), -0.34 (SF-12 role emotional 2), -0.38 (SF-12 social functioning), with physical dimension of work ability at 0.22 and mental dimension of work ability at 0.27 (Table 2). These associations are mirrored in the values of AUC in Tables 3 and 4.

**Table 2:** Rank Correlations (Kendall Tau b) with BDI-V

Variable	Males (*n* = 2,552)	Females (*n* = 2,860)
Work ability - physical^a^	0.21*	0.22*
Work ability - mental^a^	0.30*	0.27*
SF-12 role emotional 1	-0.37*	-0.39*
SF-12 role emotional 2	-0.35*	-0.34*
SF-12 social functioning	-0.35*	-0.38*

^a^reversed coding for Work ability, *p < .01

**Table 3:** BDI cut-off score, sensitivity, 1 – specificity, min-max, AUC for different criteria for male (*n* = 2,552)

Criterium	BDI-V Score	SENS	1 - SPEC	min- max	AUC
Work ability - physical^a^	≥ 20	0.64	0.36	0.36	0.68
Work ability - mental^a^	≥ 20	0.65	0.35	0.35	0.72
SF-12 role emotional 1	≥ 24	0.73	0.25	0.27	0.81
SF-12 role emotional 2	≥ 24	0.75	0.26	0.26	0.81
SF-12 social functioning	≥ 23	0.71	0.27	0.29	0.79

^a^reversed coding for Work ability

**Table 4:** BDI cut-off score, sensitivity, 1 – specificity, min-max, AUC for different criteria for female (*n* = 2,860)

Criterium	BDI-V Score	SENS	1 - SPEC	min- max	AUC
Work ability - physical^a^	≥ 23	0.59	0.40	0.40	0.65
Work ability - mental^a^	≥ 27	0.72	0.29	0.29	0.78
SF-12 role emotional 1	≥ 28	0.74	0.26	0.26	0.81
SF-12 role emotional 2	≥ 28	0.70	0.28	0.30	0.80
SF-12 social functioning	≥ 26	0.71	0.30	0.30	0.79

^a^reversed coding for Work ability

The min-max principle points to a BDI-V-cut-off between 20 and 24 for male and between 23 and 28 for female respondents. The resulting cut-offs depend on the criteria and the corresponding sensitivities ranges: They are between 0.64 and 0.75 for males and between 0.59 and 0.74 for females (Tables 3, 4). Specificity ranges between 0.64 and 0.75 for males and between 0.60 and 0.74 for females. Female respondents have higher BDI-V cut-offs for all criteria.

## Discussion

The BDI was primarily constructed as an instrument for use in clinical settings. By contrast, the applied version BDI-V was tested within a general population [16]. To the best of our knowledge this is the first time that the validity of the instrument has been investigated within a general working population.

The sample of the current study comprises workers who – despite possible impairments to health and functioning – are still employed. This is an important difference to those studies which include clinical samples of participants who are not at work for health reasons. Moreover, the selected sampling frame also had an influence on the decision to use work ability and functioning as relevant criteria for a validation. Our strict focus on employees is a major difference to the studies of Schmidt et al. [13, 16].

The distribution of BDI values differs between a clinical setting and a general working population. In a clinical setting there is mainly a need to differentiate between moderate and severe types of depressive episodes. In contrast, high depression scores are less common among employees. Furthermore, there is some evidence that the BDI provides more detailed information about higher severity levels than other depression scales [21].

Beyond the distributional aspects, there is an important difference between the application of the BDI and an assessment of major depression by a clinical interview. The latter addresses depressive symptoms along with questions towards impairment or functioning. Hence, the association between depressive symptoms and impairment is part of the diagnosis. This association is not known for the BDI-V, it is rather an assumption which formed a focus of the current study. Evidence for this association is given by rank correlations with five indicators and their corresponding AUC values, confirming the criterion referenced validity of the BDI-V. However, these results give no indication on how to classify persons as impaired or not impaired on the basis of a particular cut-off. A cut-off value given for a revised form of the original BDI is a score of 18 [22]. But a direct comparison of scores between the BDI and the BDI-V is not possible due to the different ranges of the two instruments. The revised BDI contains the 21 items of the original BDI and the sum scores cover a range from 0 to 63. The BDI-V contains 20 items and the range for scores goes from 0 to 100. In both cases the relevant cut-off value is contained within about the first third of the range, which indicates a clinically relevant depression in the framework of a validation of the revised BDI (BDI-II [23]). The selection of an appropriate cut-off value for depressive symptoms as assessed by the BDI-V depends firstly on their relation to general functioning and work ability and secondly, on the negative consequences of low sensitivity or specificity. If sensitivity is too low, there is a higher risk for misclassification by overlooking relevant impairments in the general functioning and work ability of employees. Otherwise, low specificity would result in employees with moderate depressive symptoms being classified as impaired even though they are not. Thus we are aware that methods used to determine a cut-off value for a meaningful dichotomy in reference to the association of depressive symptoms with general functioning and work ability should be both sensitive as well as specific. We chose the min-max principle as an appropriate method for finding an optimal cut-off value by minimizing both types of misclassification [20].

One of the most striking results of our study was the difference in the cut-off-values between women and men, with higher values for women. This might be related to the observation of Schmitt et al. [16] that women in general have a higher average level of BDI-V scores than men. Higher levels of depression in women have also been reported in several other studies using different instruments to measure depressive symptoms (e.g., [24–26]). The higher threshold for women could be the result of a tendency for symptom aggravation in women or a tendency for underreporting of depressive symptoms in men. An alternative explanation could be that women and men did report their depressive symptoms accurately but the coping strategies for depressive symptoms are more efficient in women than in men. In this case, similar levels of depressive symptoms in women and men would result in less impairment for women. A third explanation could be that women and men reported differently on the scales of general functioning and work ability. Then women would have a tendency to overestimate or men to underestimate their general functioning and work ability at a given level of depressiveness. All these explanations would lead to higher cut-off values in women than in men and have to be studied further in future investigations.

The overall response rate of our investigation was quite low at 27.3 %. However, a selectivity analysis of our study sample was carried out to investigate possible sources of bias. In comparison to the sampling frame of all employees subject to social security contributions, only minor differences were found in 16 crucial socio-demographic variables [27]. It is therefore likely that our responders are highly representative of all participants in the sampling frame selected for this study. Some limitations to the external validity of our investigation should be mentioned. One serious limitation is that only two age groups (1959, 1965) were included in our validation study. Therefore our results regarding cut-off values may be restricted to these middle-aged employees, meaning that these cut-off values might be not comparable to those of other age groups. This assumption, however, should be investigated by future studies. Another limitation is that our working population includes only employees subject to social security contributions. Although this applies to the vast majority of German employees, we can only hypothesize about the situation of civil servants, self-employed persons and freelancers. Furthermore, missing values could have introduced some sort of bias in our results. As the percentage of missing values, especially in the BDI-V, was not negligible in our data set, selection bias cannot be excluded. This is due to the possibility that the percentage of non-responders is either positively or negatively associated with the amount of depressive symptoms. Last but not least this is an exploratory data analysis. These considerations should be addressed in future investigations.

## Conclusion

It is our view that the question of whether higher BDI-V values indicate a reduction in general functioning and work ability as a consequence of a depressive mood is highly relevant in a work setting: When exceeding a certain threshold, the impairment of general functioning in the daily lives and work ability of employees may lead to a loss of productivity, higher absenteeism and higher retirement rates. This is especially a challenge for ageing societies as is the case in Germany [28]. Moreover, the use of cut-off values in the BDI-V for indicating relevant impairments in functioning and work ability is also interesting at an individual level, e.g. within the context of examinations by occupational physicians. Lastly, a more efficient assessment of depressive symptoms by means of validated instruments and the availability of cut-off values would have great practical value in the fields of occupational research and public health.

The current study focussed on a subset of indicators for functioning and work ability. The selection was predetermined by the availability of adequate instruments and variables. However, there are other options in the choice of instruments and variables beyond those realised in this study. This leads to the question of how these associations and cut-off values would be related when other indicators are simultaneously considered. The use of other indicators in further replication studies for the purpose of establishing consistency would be an important additional step in the validation of the BDI-V.
